# Post-Appendectomy Intra-Abdominal Abscess in Children with Perforated Appendicitis: A Narrative Review

**DOI:** 10.3390/medicina62040686

**Published:** 2026-04-03

**Authors:** Ciprian-Ioan Borca, Alexandru Cristian Cindrea, Madalin-Marius Margan, Roxana Margan, Alexandru Alexandru, Ovidiu Alexandru Mederle, Vlad Laurentiu David

**Affiliations:** 1 Doctoral School, “Victor Babeș” University of Medicine and Pharmacy, E. Murgu Square, No. 2, 300041 Timisoara, Romania; borca.ciprian@umft.ro (C.-I.B.); alexandru.alexandru@umft.ro (A.A.); 2 Department of Pediatric Surgery and Orthopedics, “Victor Babeș” University of Medicine and Pharmacy, 300041 Timisoara, Romania; david.vlad@umft.ro; 3 Emergency Department, Emergency Clinical Municipal Hospital, 300254 Timisoara, Romania; mederle.ovidiu@umft.ro; 4 Discipline of Public Health, Department of Functional Sciences, “Victor Babeș” University of Medicine and Pharmacy, 300041 Timisoara, Romania; 5 Centre for Translational Research and Systems Medicine, Faculty of Medicine, “Victor Babeș” University of Medicine and Pharmacy, 300041 Timisoara, Romania; 6 Department of Microbiology, Discipline of Hygiene, “Victor Babeș” University of Medicine and Pharmacy, 300041 Timisoara, Romania; roxana.margan@umft.ro; 7 Center for Studies in Preventive Medicine, “Victor Babeș” University of Medicine and Pharmacy, 300041 Timisoara, Romania; 8 Department of Surgery, “Victor Babeș” University of Medicine and Pharmacy, 300041 Timisoara, Romania

**Keywords:** appendicitis, peritonitis, cost efficiency, health system impact, complications, postoperative complications, anti-bacterial agents, drainage, child

## Abstract

Post-appendectomy intra-abdominal abscess (PAA) is a common and problematic complication in children with perforated appendicitis, contributing to prolonged hospitalization, readmissions, and increased healthcare costs. Despite advances in surgical and antimicrobial management, substantial heterogeneity persists in definitions, risk stratification, and treatment strategies. This narrative review aims to synthesize current evidence regarding the pathophysiology, risk factors, diagnostic pathways, clinical impact, and therapeutic approaches to PAA in the pediatric population. PAA occurs predominantly after perforated appendicitis and reflects persistent contamination and fibrin-driven loculation within the peritoneal cavity. Established predictors include fecalith presence, higher perforation severity, and elevated inflammatory markers. Diagnosis is typically established during the second postoperative week using ultrasound as first-line imaging. Management strategies vary widely, ranging from antibiotics alone to percutaneous or surgical drainage. PAA significantly increases length of stay, need for invasive procedures, and healthcare expenditure. In conclusion, PAA remains a clinically significant complication in pediatric perforated appendicitis. Standardized definitions, validated predictive tools, and high-quality trials are urgently needed to harmonize management, optimize outcomes, and reduce variability in care.

## 1. Introduction

Acute appendicitis is the most common and costly abdominal surgical emergency in childhood, and it remains one of the most common conditions in the pediatric emergency departments and surgical services [1,2]. Post-appendectomy intra-abdominal abscess (PAA) was defined as a walled-off intra-abdominal collection of purulent fluid occurring after appendectomy, identified on ultrasound, CT, or MRI, and associated with clinical and laboratory evidence of infection [3]. Clinically significant PAAs are important because they often trigger repeat imaging, prolonged or scaled antibiotics, drainage through interventional radiology or reoperation, emergency department revisits and readmissions, increasing patient morbidity and adding supplemental burden upon the health-system [1,4,5].

Globally in 2021, appendicitis incidence was 214 per 100,000, corresponding to ~17 million new cases (13.8–21.6 million), while mortality remained measurable at 0.358 per 100,000 (95% UI 0.311–0.414) with marked regional variation (1.01 per 100,000 in Central Latin America vs. 0.054 per 100,000 in high-income Asia Pacific) [6]. In children, severity at presentation is a key determinant of outcomes: a large U.S. administrative analysis (2001–2015) found rising perforation rates from 317.5 to 457.7 per 1000 pediatric appendicitis cases, translating to ~25,000 pediatric perforations annually, reinforcing the importance of timely diagnosis and treatment [7]. PAA has an incidence ranging between 1% and 24%, depending on appendicitis severity and surgical approach [3]. About 92% of these cases were complications of complex appendicitis.

These complications have a high burden on the health systems, substantially increasing the hospitalization stay (8.9 days vs. 4 days in cases of noncomplicated appendicitis), revisit rate (22.9% vs. 8.9%) and higher hospital costs ($32,282 vs. $13,296) [2]. PAA patients require additional interventions (for example, about 53% required percutaneous abscess drainage [4]). Additionally, these patients have 6.1 excess hospital bed-days, accounting for $11,809 (66%) of the total cost excess [4].

Even in modern cohorts, the incidence and cost of PAA remains high. Furthermore, complex analysis of the literature is difficult, as standardized nomenclature and detection strategies are lacking. Collective efforts have been made to create appropriate standardization tools for predicting potential complications of acute appendicitis (e.g., the National Surgical Quality Improvement Program Pediatric (NSQIP) [1]). Evidence on tools that predict PAA—both at presentation and after surgical intervention—remains limited. Current management is therefore characterized by substantial variability across institutions, and the selection criteria guiding different therapeutic strategies are inconsistently defined and rarely reported in a systematic manner. The aim of the present narrative review is to synthesize the available evidence, clarify what is currently supported by data, and describe key knowledge gaps that should be addressed to enable more consistent risk stratification and, ultimately, improve the quality of care for patients with complicated acute appendicitis.

Given the narrative design and the heterogeneity of the available literature, the statements in this review should be interpreted as evidence-informed summaries rather than formal guideline-level recommendations.

## 2. Literature Search

This study represents a narrative review of the current literature on PAA in children with perforated appendicitis. A structured search was performed in PubMed from database inception through to 31 January 2026. The search strategy combined Medical Subject Headings (MeSH) and free-text terms, including the following: “Appendicitis, Perforated”, “Appendectomy”, “Abdominal Abscess”, “Postoperative Complications”, “Child”, “Anti-Bacterial Agents”, and “Drainage”, along with related keywords such as “intra-abdominal collection” and “percutaneous drainage”. Eligible studies were reports relevant to the epidemiology, pathophysiology, predictors, diagnosis, outcomes, or management of PAA after appendectomy in children. When pediatric evidence was limited for a specific mechanistic or therapeutic question, selected adult or mixed-population studies were included only as indirect contextual evidence and are explicitly identified as such in the text. Reports not focused on postoperative PAA, studies dealing exclusively with non-operative appendicitis management without appendectomy, and articles without extractable relevance to the review questions were not emphasized in the synthesis.

Records were screened by title/abstract followed by full-text review for relevance. Priority was given to systematic reviews, randomized or prospective studies, multicenter pediatric cohorts, and contemporary guidelines, while older landmark publications were retained when necessary for historical or mechanistic context. Reference lists of included studies were also screened manually for additional relevant reports. Because this is a narrative review, no formal risk-of-bias tool or quantitative synthesis was applied; accordingly, the conclusions are presented as evidence-informed interpretations of the heterogeneous literature rather than formal guideline recommendations.

This review was conducted following the principles outlined by the SANRA checklist for narrative reviews.

## 3. Short History

**Figure 1:**
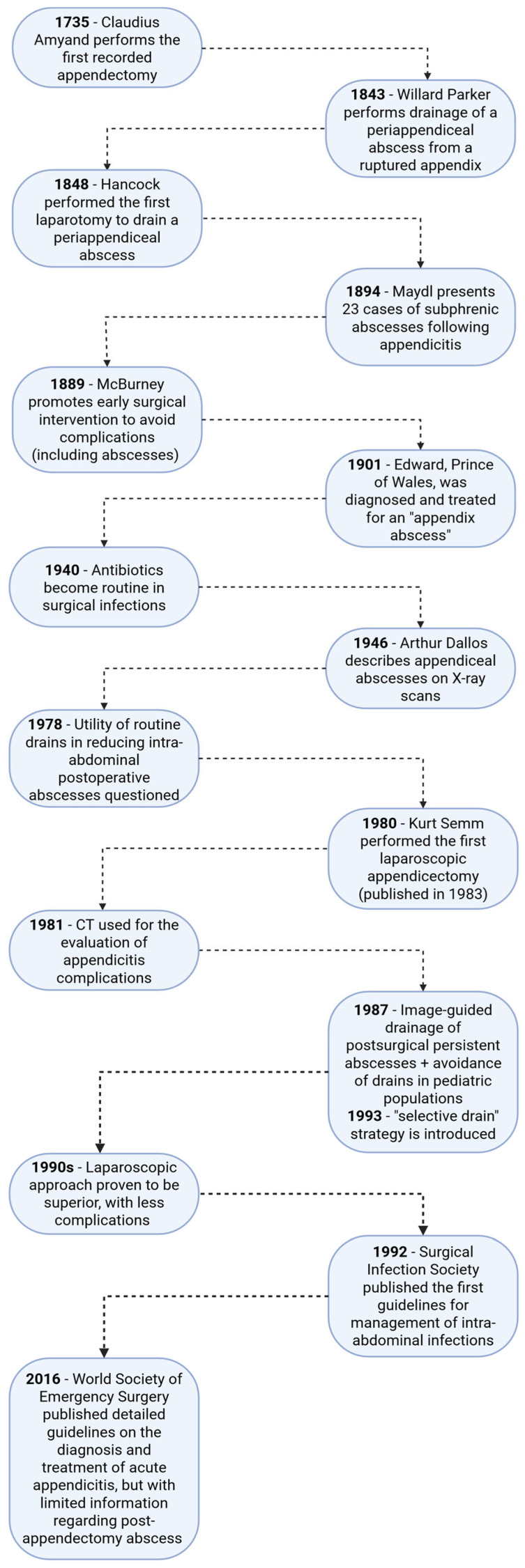
Historical marks in the study and treatment of acute appendicitis complicated with post-appendectomy abscess [8,9,10,11,12,13,14,15,16,17,18,19,20,21,22,23,24,25,26]. Created in https://BioRender.com. Created in BioRender. Cindrea, A. (2026) https://BioRender.com/mwe79e5.

Acute appendicitis became a distinct clinicopathologic entity in the late nineteenth century, when Fitz demonstrated that inflammation previously labeled as “typhlitis” originated from the appendix and that delayed recognition led to perforation, peritonitis, and abscess formation, thereby establishing the biological basis for postoperative septic complications such as PAA [8]. In the mid-twentieth century, postoperative intra-abdominal abscesses were a major source of morbidity and mortality after appendectomy, and early reports of recurrent appendiceal abscess due to residual appendiceal tissue highlighted that PAA could occur despite surgical intervention [8,9]. The introduction of broad-spectrum antibiotics reduced mortality but did not eliminate PAA, leading to decades of debate over preventive strategies such as routine intraperitoneal drainage. The latter was, eventually, shown to offer no benefit, rather causing potential harm [5,10,11,12,13]. Contemporary international guidelines now frame PAA as a common but manageable complication of perforated appendicitis, emphasizing selective drainage, short-course antibiotics, and standardized, evidence-based pathways, while ongoing uncertainty underscores the need for improved risk stratification and uniform definitions in pediatric populations [14,15,16]. Some milestones in the evaluation and treatment of PAA are presented in Figure 1.

## 4. Pathophysiology

**Figure 2:**
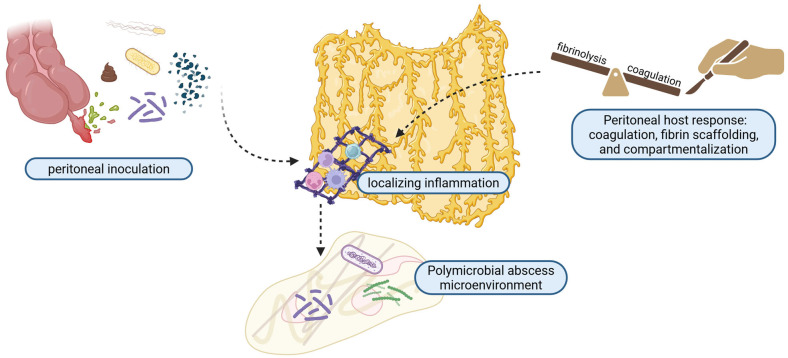
Pathophysiology of intra-abdominal appendiceal abscesses. Perforation permits peritoneal inoculation with a polymicrobial enteric load (aerobes and anaerobes) together with materials such as pus, necrotic debris, fibrin, and stool micro-particles, and, sometimes, a dropped appendicolith. The omentum and adjacent viscera attempt to localize inflammation, physically and immunologically isolating the contaminated area. Concurrently, the peritoneal host response shifts toward coagulation, with fibrin scaffolding favored over fibrinolysis, which helps contain infection early but also promotes loculation by trapping organisms and contaminated fluid in poorly drained pockets. When bacterial clearance and source control are incomplete, these fibrin- and adhesion-bounded spaces evolve into a polymicrobial abscess microenvironment, providing a protected niche that supports persistence and maturation into a clinically apparent intra-abdominal abscess [28,29,30,31,32,33,34,35,36,37]. Created in BioRender. Cindrea, A. (2026) https://BioRender.com/ihncn93.

In children, PAA occurs disproportionately after perforated (complicated) appendicitis because perforation converts a primarily intraluminal infection into secondary peritonitis with gross bacterial and particulate contamination of the peritoneal space (Figure 2) [27]. Upstream events typically begin with appendiceal luminal obstruction (frequently fecalith/appendicolith or lymphoid hyperplasia), which increases intraluminal pressure, compromises venous and lymphatic outflow, and progresses to ischemic transmural necrosis and perforation [27,28,29]. Perforation releases not only bacteria but also pus, necrotic tissue, fibrin, and fecal micro-particulates (and sometimes an appendicolith), and these particulate substrates can serve as scaffolds/nidi that favor bacterial adherence, persistence, and later abscess maturation despite appendectomy [30,31].

The peritoneum initially limits systemic spread through mechanical clearance via diaphragmatic lymphatic stomata, rapid recruitment of phagocytes, and sequestration of organisms within fibrinous matrices [33,35]. During peritonitis and surgical manipulation, the local balance shifts toward coagulation: fibrinogen extravasates into the cavity, tissue factor–thrombin signaling converts it into fibrin, and mesothelial upregulation of plasminogen activator inhibitors suppresses fibrinolysis, allowing fibrin to persist and organize [32,33,34]. This fibrin response is biologically “double-edged”, because it helps contain bacteria early, resulting in partitions of contaminated fluid. Therefore, bacteria are protected from phagocytes and antibiotics, enabling unopposed proliferation within an evolving cavity. Experimental observations further support the causality of coagulation biology in peritoneal infection control. Fibrinogen-dependent mechanisms drive rapid intraperitoneal bacterial clearance, whereas disruption of fibrin/fibrinogen availability worsens outcomes and can alter abscess formation [32,33]. In parallel, injury and activation of peritoneal mesothelial cells promote mesothelial-to-mesenchymal transition with extracellular matrix production (including collagenous scaffolding), which contributes to the fibrous pseudocapsule that characterizes a mature abscess [34].

Pediatric anatomy modifies these containment dynamics because the greater omentum is relatively thin and less developed in younger children, reducing early physical sequestration and allowing wider distribution of infected material to dependent spaces (notably pelvis and paracolic gutters) before later re-localization into discrete collections [27,37,38,39]. Omental “milky spots” (clusters of macrophages, B-lymphocytes, T-lymphocytes, mast cells and stromal cells) support bacterial clearance and act as a mobile immunologic platform that adheres to inflamed viscera [36]. Host inflammatory signaling is required to build the cellular architecture of an abscess, and experimental work indicates that neutrophil-derived IL-1β can be a key amplifier of recruitment and organization at the infectious focus [40,41]. Clinically, perforated appendicitis in children is associated with heightened systemic and peritoneal cytokine activity (including TNF-α and IL-6), reflecting the magnitude of contamination and the intensity of innate immune activation that precedes abscess maturation [42,43].

Microbiologically, PAA is usually polymicrobial and reflects distal gut flora, with frequent recovery of enteric Gram-negative aerobes (e.g., *Escherichia coli*) together with anaerobes such as *Bacteroides fragilis* (and, variably, *Fusobacterium* spp. and mixed streptococci) [44,45]. Synergy between aerobes and anaerobes is central to abscessing, because oxygen consumption by aerobes lowers redox potential and creates conditions permissive for obligate anaerobes, and anaerobic beta-lactamase production can provide “cross-protection” to co-resident aerobes under antibiotic pressure [46]. A biphasic model helps explain clinical evolution: facultative aerobes are major drivers of early peritonitis/systemic inflammatory response, while anaerobes become dominant within the mature, hypoxic abscess niche.

The “postoperative timing paradox” is explained by the persistence and evolution of residual contamination rather than truly new infection: appendectomy removes the appendix but may leave contaminated micro-environments in dependent recesses where fibrin-driven loculation continues until a mature cavity becomes radiologically apparent (often beginning around postoperative day 5 and commonly within the first 1–2 weeks) [31,47,48].

## 5. Risk Factors and Predictors for Developing PAA

Younger children are, statistically, more likely to present with perforated appendicitis [49]. Conversely, the role of age as an independent risk factor for developing intraabdominal abscesses remains controversial, with some studies reporting older age as a risk factor [50,51], while others report no influence of age in the risk of developing PAA [51]. While males exhibit a slightly higher overall incidence of appendicitis (1.4:1) [52], some studies have identified female sex a potential risk factor [51]. The body mass index is another risk factor for PAA [50], but BMI-based classifications do not reliably predict complicated appendicitis [53]. Regarding the clinical vignette of the patients, those who presented with diarrhea were more than three times more likely to develop PAA [50,54], while patients that presented with bowel obstruction or severe ileus had a six times higher risk [5].

The most consistently supported predictors were the presence of a fecalith and greater perforation severity. Henry et al. [54] found that intraoperative fecaliths are associated with an 8.77 higher rate of developing PAA, while Son et al. [48] found a risk of 5.55 times. Higher values of the American Association for the Surgery of Trauma severity grade are associated with high rates of PAA (OR 5.9) [48].

Higher inflammatory burden (characterized by high WBC counts, high neutrophil to lymphocyte ratio (NLR), high C reactive protein (CRP) values) upon admission is associated with higher abscess rates [5,48,55]. CRP to lymphocyte ratio exhibits high discriminatory ability, with an AUC of 0.772 (95% CI: 0.733–0.812) for complicated appendicitis, but its value in detecting PAA specifically is yet to be tested [56]. Lodwick et al. found a 4.46 higher risk of developing PAA in patients with lymphopenia and proposed a management algorithm for patients diagnosed with appendicitis [57].

Postoperative fever on the third day of admission is inconsistently associated with higher abscess incidence [50,58]. Children with a regular postoperative diet seem to have a lower risk of developing PAA [54].

**Figure 3:**
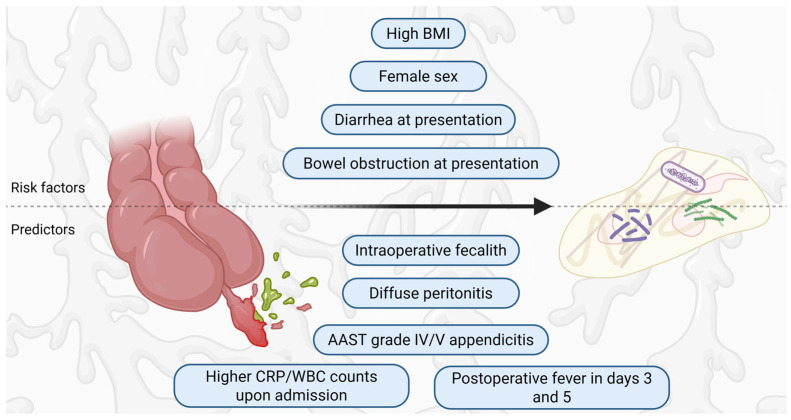
Risk factors and predictors for developing post-appendectomy abscesses. Created in BioRender. Cindrea, A. (2026) https://BioRender.com/bo4pdf7.

The risk factors and predictors of PAA in children are summarized in Figure 3. Overall, reliable predictive factors for PAA are lacking, making it difficult to predict which patients will develop this complication.

However, most demographic or presentation-based risk factors (e.g., age, sex, BMI, diarrhea, ileus) show inconsistent associations across cohorts, and several likely reflect confounding by disease severity or delays to diagnosis rather than independent predictors of PAA. In contrast, intraoperative markers of disease burden—particularly appendicolith/fecalith and higher perforation grades—appear more reproducible and may be more useful anchors for pragmatic risk stratification. Methodological limitations include predominantly retrospective single-center designs, heterogeneous definitions of abscess (true abscess versus sterile postoperative collection), and detection bias introduced by routine imaging protocols. Taken together, the evidence suggests that currently proposed prediction models require external validation before they can reliably guide imaging frequency or early escalation of therapy.

Intraoperative factors may contribute to the risk of PAA formation, but their effect appears less consistent. In pediatric complicated appendicitis, laparoscopic appendectomy has not been shown to increase intra-abdominal abscess formation compared with open appendectomy in contemporary meta-analysis, despite earlier concerns. Similarly, peritoneal irrigation has not demonstrated a clear benefit over suction alone in preventing PAA and may prolong operative time. Current evidence does not support routine extensive irrigation solely for abscess prevention, and the intraoperative strategy should instead prioritize effective source control with the least additional tissue burden [59,60].

## 6. Diagnostic Pathway

In children who have been operated on for perforated appendicitis, PAA is a major driver of postoperative morbidity. In contemporary pediatric cohorts of perforated appendicitis, PAA is most commonly observed during the late first or second postoperative week, with a median time to diagnosis of about 8 days (with variable IQRs of 4–29 days) [3,5,61]. This timing is important because early postoperative imaging may reveal small fluid collections (e.g., seromas, hematomas, irrigation fluid) that are sterile, self-limited (usually resolves in about 5–7 days), or otherwise clinically insignificant and therefore do not necessarily represent true abscesses [62].

Suspicion may rise when postoperative recovery stalls or reverses, particularly in the presence of persistent or recurrent fever, abdominal pain, gastrointestinal dysfunction/ileus, or failure to resume a diet after initial improvement [5,50,58,62,63].

Ein et al. [63] studied the role of routine abdominal imaging in predicting PAA formation at postoperative day 5. The authors found that about 82% of the patients were asymptomatic and had a PAA less than 5 cm. Those patients required no further treatment. Out of those, 6% returned within a week, presenting characteristic symptoms, and had a larger abscess. The authors concluded that routine abdominal imaging on postoperative day 5 did not improve prediction compared with clinical evaluation.

According to guidelines, in children with suspected intra-abdominal abscess, ultrasound is an appropriate initial imaging test, with CT or MRI reserved for negative, equivocal, or nondiagnostic ultrasound when clinical suspicion persists [62]. This staged strategy is particularly relevant after appendectomy, where indiscriminate early imaging risks overdiagnosis and unnecessary escalation to antibiotics or drainage.

Non-contrast MRI utilizing fluid-sensitive and DWI sequences can be used to identify drainable PAAs, therefore facilitating the triage between conservative management vs. drainage without administration of contrast and exposure to ionizing radiation and with less costs [64,65,66]. MRI can also help identify safe drainage pathways, which may be particularly useful for pelvic abscesses [62,64,66].

Labeling heterogeneity persists across studies and thresholds are not directly interchangeable. It is important to make a difference between fluid collections without an enhancing ring and true abscesses. The classification of PAA varies, with authors using the criteria of size (determined using imaging studies) in order to assess the treatment success ratio. Some authors consider a diameter threshold of 3 cm for large abscess, while others use the 4, 5 or even 6 cm threshold [67,68]. Van Amstel et al. [61] used a three-level classification: small (below 3 cm), medium (3–6 cm) and large (above 6 cm). On a two-dimensional scale, large PAAs are those which exceed 20 cm^2^ [69]. Some authors used a volumetric classification of the abscess, with thresholds for drainage of 100 cm^3^ [70], or even a weight-adjusted volumetric cut-off of 2 mL/kg [71]. These disparate size, surface-area, and volume cutoffs complicate direct comparison of treatment strategies and likely explain why recommendations sometimes appear contradictory. Diameter is simple but can misrepresent irregular or multiloculated abscesses; conversely, volumetric metrics (including weight-adjusted thresholds) may be more physiologically appropriate for children but are currently supported mainly by observational data and require prospective validation. Further studies are required to determine the most appropriate classification paths in order to ensure appropriate management of the patient. Additional data regarding PAA classification can be found in Table 1.

Accordingly, size alone is not sufficient to guide management; clinical condition, systemic illness, number and complexity of collections, anatomical accessibility, and early response to treatment remain equally important determinants of whether conservative therapy or drainage is more appropriate.

## 7. Impact of PAA on Patient’s Outcomes

In children operated on for perforated appendicitis, PAA is a major driver of postoperative morbidity and healthcare utilization [4,54,72].

### 7.1. Antibiotic Regimen

PAA commonly necessitates a new therapeutic antibiotic course (escalation in duration and coverage) after the abscess is identified, frequently representing escalation beyond the initial postoperative regimen used for complicated appendicitis [4,73,74,75]. Antibiotics are the primary treatment for PAA, with most studies reporting high success rates (86–97%) regardless of size, but the evidence remains heterogeneous [68]. The presence of PAA necessitates restarting or prolonging antibiotic regimens post-discharge in many cases [76]. Children with PAA are less likely to respond to initial non-operative management than patients who developed a phlegmon [77].

Patients often require extended intravenous antibiotic therapy, with studies showing a median duration of 12–15 days for intravenous treatment, or longer for larger abscesses (>6 cm) [68]. Evidence suggests a profound lack of consensus regarding the optimal duration; some institutions follow clinical parameters alone, while others adhere to rigid fixed-day courses. Research at Phoenix Children’s Hospital is currently evaluating the equivalence of 4-day versus 8-day courses following the drainage of a post-surgical abscess, attempting to balance therapeutic efficacy against the risks of multi-drug resistance [78].

### 7.2. Invasive Interventions

About 40% of these abscesses necessitate re-operation or invasive intervention, including percutaneous or surgical drainage, particularly when the abscess is large, multiloculated, or fails to respond to conservative management [3,48,61].

For re-operation, while conservative management with antibiotics succeeds in most cases, approximately 3–13% of PAA patients require re-operation for complications such as retained appendicoliths, small bowel obstruction, or recurrent abscess [48].

Invasive procedures, including percutaneous drainage or surgical re-intervention, are needed in 30–40% of cases, particularly for larger abscesses or treatment failures. Interventional radiology through percutaneous abscess drainage (PCD) has largely superseded open surgical re-intervention as the first-line therapy for localized, unilocular abscesses [79]. Technical success rates for PCD are high, often cited at 84% to 90%, and the procedure significantly reduces the anatomical trauma associated with a second laparotomy [80].

### 7.3. Length of Hospitalization

Post-appendectomy abscesses are associated with a markedly prolonged length of hospitalization; children treated non-invasively still have longer stays than those without PAA, while those requiring drainage often have the longest inpatient courses.

One pediatric cohort reported median hospital stays of about 7 days in non-invasive treatment versus 17 days in invasive-treatment groups, underscoring the resource burden of abscess-related care [3]. In the analysis by Fike et al. [72], total length of stay increased from 5.1 days (no abscess) to 11.6 days (postoperative abscess) among children with perforated appendicitis, which roughly translates to an additional inpatient week. Ferguson et al. [4] similarly estimated 6.1 excess hospital bed days attributable to postoperative abscess after adjustment for confounding. In another study, mean initial hospitalization time was 14 ± 7.96 days when abscess was diagnosed during the index admission, while mean rehospitalization was 11.9 ± 7.13 days among those readmitted for abscess [74].

### 7.4. Hospital Readmission Rate

Readmissions are a frequent outcome, with 57–60% of PAAs diagnosed after initial discharge, leading to readmission within a median of 6–11 days for further management. Readmission rates for PAA-related issues range from 3.6 to 11.6%, with no significant reduction from post-discharge antibiotics, highlighting the abscess as a key driver of unplanned returns to hospital [48,68,69,76,81]. Readmission rate did not vary with abscess size (27 vs. 26 patients, *p* = 0.786) [68]. PAAs were more frequent in patients treated conservatively compared with the drainage group (33% vs. 10%) [5].

### 7.5. Return to School Time

Return to school is delayed in children with PAA due to extended recovery periods, typically occurring 7–10 days after discharge or when the child feels comfortable and no longer requires pain medication or drainage, compared to 1–2 days for uncomplicated appendectomy. Complications like abscess formation prolong overall convalescence, impacting the resumption of normal activities including school attendance for up to 2–3 weeks or more in severe cases [82].

### 7.6. Mortality

Mortality associated with post-appendectomy PAA in children with perforated appendicitis is extremely rare in the modern era, with most contemporary pediatric series reporting zero deaths directly attributable to this complication. Large multicenter studies, including data from NSQIP involving over 28,000 appendectomies, reports an overall 30-day mortality rate of 0% or only 2 deaths (0.007%), despite postoperative sepsis occurring in 0.4% of cases [83,84].

## 8. Therapeutic Approach

The therapeutic goals in pediatric PAA are rapid control of infection, prevention of sepsis progression, minimization of invasive procedures when safe, and antibiotic stewardship (appropriate spectrum, dose, and duration) [16,61,85]. Management should be driven by the child’s physiologic stability (toxicity, hemodynamics, mental status), because unstable children require urgent source control and broad empiric coverage rather than prolonged watchful waiting [61,85]. A structured reassessment plan (clinical course, fever trajectory, pain, feeding tolerance, inflammatory markers) is recommended because failure of improvement often reflects inadequate source control.

From a prophylaxis standpoint, available pediatric studies suggest that routine continuation of antibiotics after discharge may not reduce subsequent PAA in selected children recovering after appendectomy for perforated appendicitis [76,86]. In practice, discharge without antibiotics may be an option for clinically improving children who meet predefined criteria, such as being afebrile for at least 24 h, tolerating oral intake, having adequate pain control, demonstrating reassuring clinical or laboratory improvement, and having reliable follow-up [87]. Because the supporting data are predominantly retrospective, this strategy should be framed as selective antibiotic stewardship in carefully chosen patients rather than as a standard approach for all postoperative patients.

There seems to be no difference in outcomes after surgical intervention for perforated appendicitis with or without antibiotics at hospital discharge. Elimination of postoperative antibiotic use could result in better antibiotic stewardship, decreased patient cost, and decreased hospital length of stay. Further research with a randomized, prospective trial is warranted [81].

Prophylactic abdominal drainage following appendectomy does not seem to reduce the incidence of PAA, but rather to increase the incidence of some postoperative complications [88].

Intra-abdominal cultures may not be routinely obtained during regular appendicitis surgery [62]. The results of the cultures taken during surgery and from the subsequent abscess seem not to overlap and the lack of antibiotic coverage of intraoperative cultures does not look to be an important factor in abscess formation [89].

Standard protocols for complex appendicitis usually target a 3-to-5-day course of intravenous (IV) ceftriaxone and metronidazole, transitioning to oral agents when the child becomes afebrile for 24 h [26]. According to the Surgical Infection Society [87], when initial therapy has failed, management should prioritize the least invasive effective source-control strategy together with an appropriate intravenous regimen, with reassessment of antimicrobial selection when resistant organisms are a concern. The guidelines also advise against unnecessarily prolonged intravenous therapy, recommending that IV antibiotics generally not be continued beyond 7 days in children with perforated appendicitis and PAA. In practice, this means that commonly used regimens such as ceftriaxone plus metronidazole may remain appropriate in many children, whereas broader empiric agents such as piperacillin-tazobactam, imipenem-cilastatin, or meropenem may be reserved for selected higher-risk situations, including severe sepsis, delayed or inadequate source control, substantial prior healthcare or antibiotic exposure, or concern for resistant organisms.

Regarding PAA, a treatment protocol in which a step-up approach for children with a PAA < 6 cm, reserving invasive drainage procedures for children in an unstable condition with signs of sepsis or septic shock, was proposed. For children presenting with large (>6 cm) PAA or multiple PAAs, initial percutaneous drainage is advised [61,86]. Other authors recommend the usage of a volumetric cutoff of 2 mL/kg to guide drainage decision [71]. When drained, abscesses larger than 100 mL have a better recovery time [90]. However, a recent study suggests that antibiotic only management can be considered regardless of abscess size, with similar therapeutic failure, complications and reoperation rates [68].

In one pediatric surgery department experience, antibiotic therapy for the abscess specifically shifted to imipenem (alone or combined with metronidazole), distinct from their cephalosporin-plus-metronidazole protocols for complicated appendicitis [74]. In a separate pediatric cohort, Dhaou et al. [75] reported a standardized post-diagnosis triple intravenous regimen (cefotaxime, gentamicin, metronidazole) with serial reassessment and discontinuation only after clinical improvement and normalization of laboratory/radiologic findings.

Further investigation, consisting of randomized controlled trials and meta-analyses, is necessary to determine a proper antibiotic regimen for this patient population.

## 9. Limitations

This review has several limitations. It is narrative rather than systematic, no formal risk-of-bias assessment was performed, and much of the pediatric evidence derives from retrospective single-center studies with heterogeneous definitions of PAA, thresholds for imaging and drainage, and outcome measures. In addition, selected adult or mixed-population studies were used for contextual or mechanistic purposes where pediatric data were sparse and should not be interpreted as directly transferable to children. These limitations should be considered when interpreting the management statements summarized here.

## 10. Conclusions

In conclusion, post-appendectomy intra-abdominal abscess remains a frequent and clinically relevant complication in children with perforated appendicitis, contributing substantially to prolonged hospitalization, readmissions, invasive interventions, and increased healthcare costs. Despite advances in surgical techniques, imaging modalities, and antimicrobial therapy, considerable heterogeneity persists in definitions, risk stratification, imaging thresholds, and treatment algorithms. Reliable predictive tools for identifying children at highest risk of PAA are still lacking, and high-quality randomized studies are needed to refine antibiotic duration, drainage criteria, and standardized classification systems. A more uniform, evidence-based framework may ultimately reduce variability in care, optimize outcomes, and lessen the burden of this complication on both patients and health systems.

## Figures and Tables

**Table 1:** Size classification of PAA according to different authors.

Authors	Study Type	N	Threshold Used	Outcome Assessed	Main Finding	Key Limitation
Collins et al., 2020 [67]	Retrospective cohort	4901	4 cm	Success of non-operative management	Supports non-operative treatment for small post-appendectomy abscesses	Not pediatric-specific; retrospective; threshold based on single maximal dimension rather than complexity or volume
van Amstel et al., 2022 [61]	Meta-analysis	1355	<3 cm, 3–6 cm >6 cm	Choice and outcome of non-invasive vs. drainage strategies	Proposed practical three-level classification, but management still depended on clinical, biochemical, and radiologic factors	Not a randomized comparison; heterogeneous treatment selection; size not used in isolation
Moreno-Alfonso et al., 2023 [68]	Case–control study	1766	≤6 cm >6 cm	Efficacy of antibiotic-only treatment	Antibiotics had high success in both groups (97% for ≤6 cm vs. 86.4% for >6 cm), although larger abscesses required longer IV antibiotics and longer stay	Single-center; retrospective; no randomization; larger abscesses still had greater resource use; size alone may not explain outcomes
Svetanoff et al., 2020 [69]	Prospective observationa	30	20 cm^2^	Need for drain placement and outcomes under institutional algorithm	Institutional algorithm limited drainage to abscesses >20 cm^2^; smaller abscesses were treated with antibiotics only	Institutional algorithm rather than externally validated threshold; area-based measure not directly comparable with diameter- or volume-based cutoffs
Bough et al., 2022 [71]	Retrospective cohort	60	2 mL/kg	Need for drainage/intervention	Suggested that 2 mL/kg is a useful objective measure for intervention and may outperform single-dimension size assessment	collection volume estimation may vary by imaging and calculation method; not all collections are equivalent in loculation/accessibility

## Data Availability

No new data were created or analyzed in this study.

## Abbreviations

The following abbreviations are used in this manuscript:

AUC	Area Under the Curve
CRP	C-Reactive Protein
CT	Computed Tomography
DWI	Diffusion-Weighted Imaging
HR	Hazard Ratio
IQR	Interquartile Range
IV	Intravenous
MRI	Magnetic Resonance Imaging
NLR	Neutrophil-to-Lymphocyte Ratio
NSQIP	National Surgical Quality Improvement Program
OR	Odds Ratio
PAA	Post-Appendectomy Intra-Abdominal Abscess
PCD	Percutaneous Drainage
TNF-α	Tumor Necrosis Factor Alpha
WBC	White Blood Cell (count)
WSES	World Society of Emergency Surgery
